# Management of recalcitrant oral pemphigus vulgaris with CO_2_ laser — Report of two cases

**DOI:** 10.4103/0972-124X.70835

**Published:** 2010

**Authors:** Ashu Bhardwaj, Monika Joshi, Deepak Sharma

**Affiliations:** *Department of Periodontics, Himachal Pradesh Government Dental College and Hospital, Shimla, Himachal Pradesh, India*

## Abstract

Laser has been used efficiently for treatment of oral lichen planus, leukoplakia, aphthous ulcers and oral manifestations of HIV. Two cases of recalcitrant oral pemphigus vulgaris that were successfully treated with CO_2_ laser are described. The patients had been treated by a dermatologist with pulse therapy of methyl prednisolone and cyclophosphamide over a period of 6 to 8 months, but the clinical course was characterized by episodes of painful flare-ups and nonresponsiveness. The patients were extremely uncomfortable with recurrent oral lesions. CO_2_ laser at low power was used to irradiate the lesions. It was shown to be effective in relieving pain and healing of lesions, with nonrecurrence. To the best of our knowledge, this is the first case report of such a treatment of oral pemphigus vulgaris. Further clinical studies are warranted to confirm efficacy and to optimize the treatment protocol.

## INTRODUCTION

Systemic steroid therapy is still the mainstay of treatment for pemphigus vulgaris; however, this treatment modality is limited by various adverse effects. To reduce the side effects, steroid-sparing agents are often used.[1] Some patients do not respond to such treatment modalities. Management of recalcitrant pemphigus vulgaris poses great problems. It has been proposed in literature that CO_2_ laser can be used to treat vesiculo-bullous lesions in the oral cavity.[2] Lasers have been used to treat leukoplakia, lichen planus and for palliative effect in aphthous ulcers and oral manifestations of HIV. We have described 2 such patients of oral pemphigus vulgaris who were not responding to systemic steroid therapy. CO_2_ laser at low power was used to relieve pain, discomfort and prevent recurrence of the lesions.

## CASE REPORTS

### Case 1

**Figure 1:**
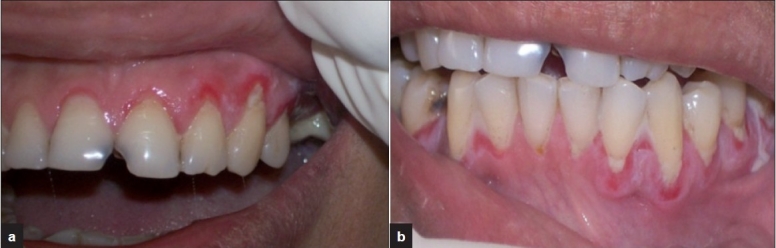
(a, b) Gingival desquamation

A 40-year-old woman presented with 6-month history of burning sensation in gums, pain while swallowing and brushing, with painful gingival erosions and desquamation [Figure 1a and b]. There were denuded, spontaneously bleeding gingival zones. The gingival margin and some areas of attached gingiva and interdental papilla were erythematous and denuded. Positive Nikolsky’s sign was present. The lesions were typical of desquamative gingivitis. The clinical picture with positive Nikolsky’s sign offered insight into the possibility of presence of a vesiculo-bullous disease.

**Figure 2:**
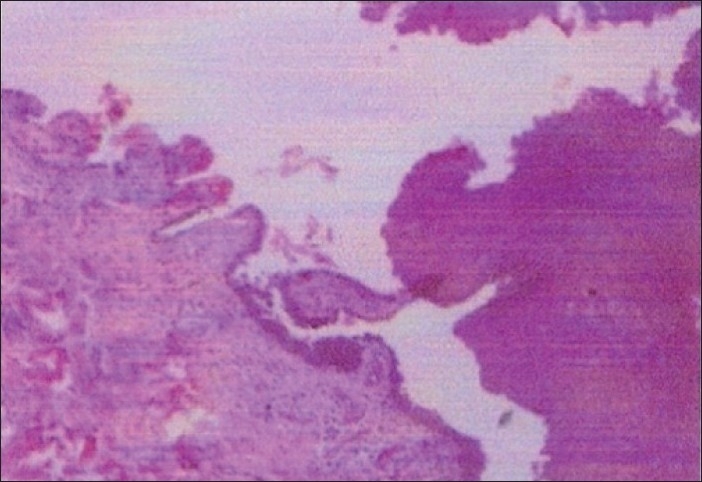
Histopathology showing suprabasal acantholysis (H and E)

She had undergone treatment for gingivitis by a local dentist but without any improvement. The medical and family histories were not significant. Extra oral examination revealed no abnormalities. A perilesional incisional biopsy was performed. Histopathological examination revealed partially denuded squamous lining with suprabasal separation and villous projections lined by basal layer. Scattered acantholytic cells were seen with underlying dense chronic inflammation [Figure 2]. Tissue for DIF (direct immuno-fluorescence) showed detached epithelium with 2+ granular squamous inter-cellular substance staining for IgG and minimal staining for C_3_· IgM was negative. A final diagnosis of pemphigus vulgaris was made on the basis of clinical, histopathologicl and DIF findings.

**Figure 3:**
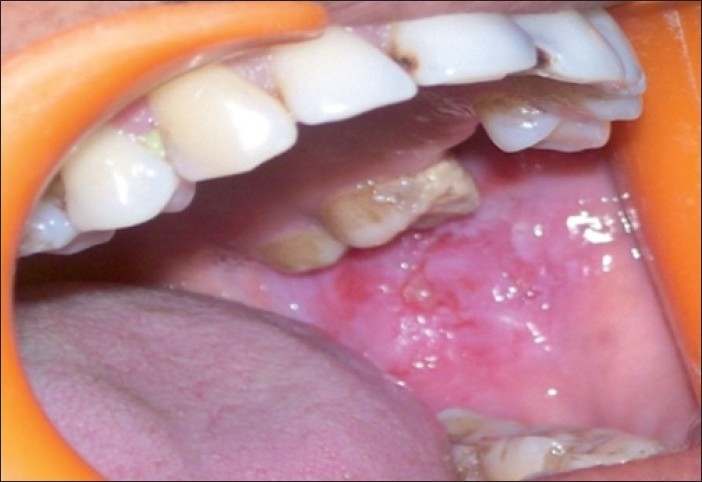
Erosions on buccal mucosa

**Figure 4:**
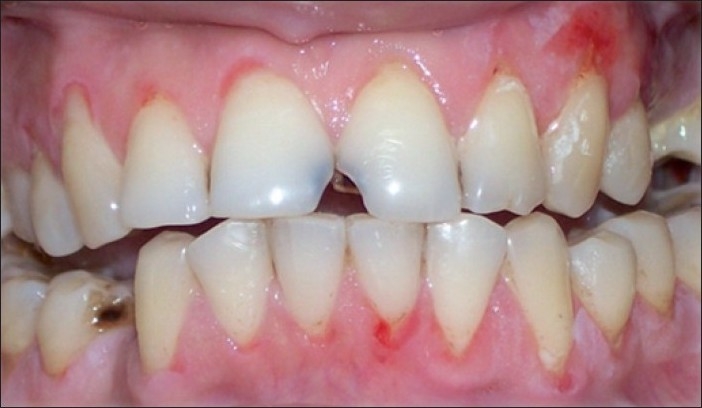
Gingival lesions visualized after 6 months of systemic steroids

The patient was treated with pulse therapy of methyl prednisolone and cyclophosphamide by a dermatologist over a period of 6 months. The disease proved to be recalcitrant to this therapy. New erosions developed on left buccal mucosa [Figure 3]. The gingival lesions were unresponsive to treatment even with addition of potent topical corticosteroids [Figure 4]. The patient was extremely distressed and depressed because of the unremitting disease activity. It has been proposed in literature that CO_2_ laser can be used for treatment of not only white lesions, premalignant lesions but also for vesiculo-bullous lesions in the oral cavity.[2] This prompted us to try therapy with CO_2_ laser.

**Figure 5:**
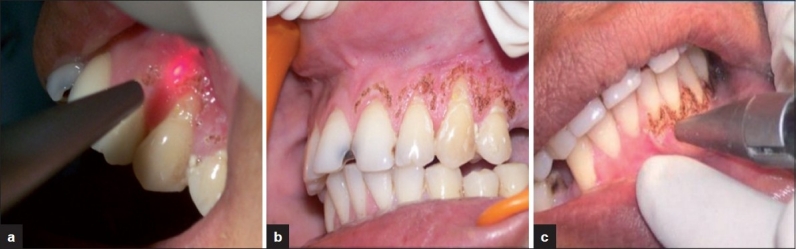
(a-b-c) CO_2_ laser irradiation

**Figure 6:**
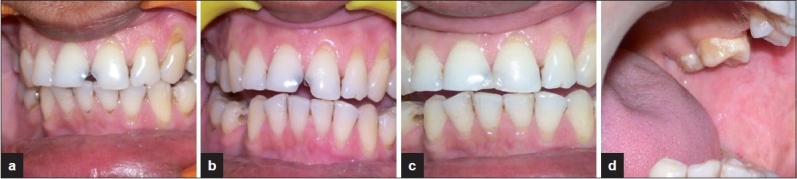
No recurrence at (a) 1-month follow-up irradiation (b) 3-month follow-up (c,d) 5-month follow-up

CO_2_ laser at 1.0-1.5 W, was used. The lesions were irradiated in a defocused mode for 5-10 [Figures 5a–c]. The patient became symptom free. Recall examinations after 1 month, 3 months and 5 months revealed complete healing of lesions [Figure 6a–d].

### Case 2

**Figure 7:**
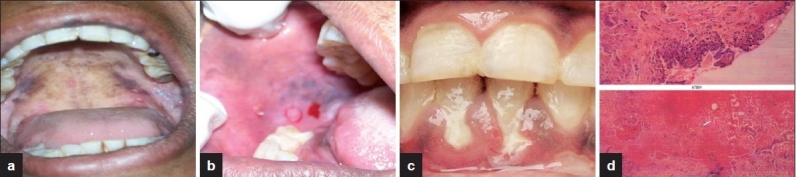
(a) Vesicles on soft palate (b) bullous lesion on buccal mucosa (c) bullous lesions on gingiva (d) suprabasal shedding of surface layers with acantholytic cells

**Figure 8:**
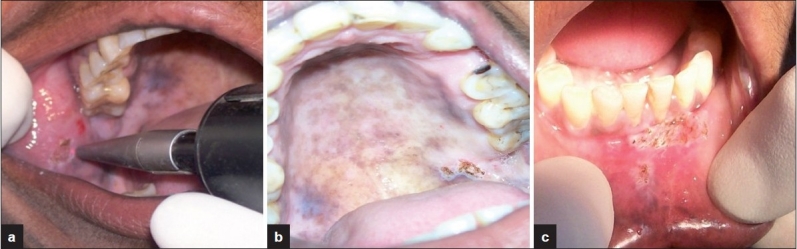
(a) CO_2_ laser irradiation (b, c) CO_2_ laser irradiated areas

**Figure 9:**
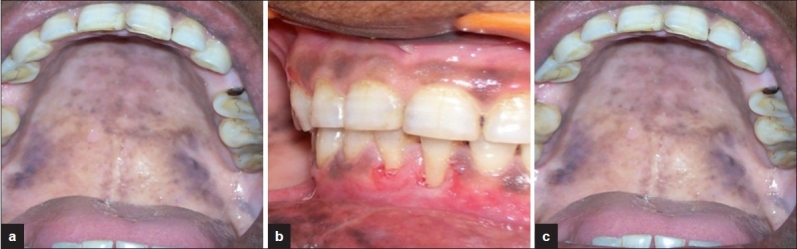
No recurrence at (a) 1-month follow-up (b) 3-month follow-up (c) lesions visualized on palate

A 50-year-old woman was referred by the Department of Dermatology for dental opinion of oral pemphigus vulgaris lesions. Fluid-filled blisters, bullae and vesicles were present on soft palate, buccal mucosa, gingiva and muco-buccal fold areas [Figure 7a–c]. The patient suffered from pain in mouth, difficulty in speaking and eating. She had visited a dentist for the same problem 3 to 4 months back and was prescribed topical steroids but there was no improvement. Medical history revealed that she had been treated for skin lesions with methyl prednisolone and cyclophosphamide for at least 7 to 8 months, but the oral lesions were not responding to the treatment and were recurrent. A biopsy was obtained. Histopathological examinations revealed suprabasal shedding of surface layers with acantholytic cells [Figure 7d]. DIF studies showed granular deposits of lgG and C3 in intercellular spaces between keratinocytes. A final diagnosis of pemphigus vulgaris was confirmed. It was decided to treat these lesions with CO_2_ laser, as for the previous patient. CO_2_ laser was used (10.6 nm wavelength continuous wave at 1.0-1.5 W) to irradiate the lesions on one side of the mouth for 5-10s [Figures 8a–c]. The patient reported no pain after treatment. The healing process was checked at 1-month and 3-month follow-up visits [Figure 9a–c]. No recurrence was seen. Subsequently other lesions were treated. The patient was symptom free.

## DISCUSSION

Systemic steroids remain the treatment of choice for pemphigus as they are both effective and capable of inducing a rapid remission. However, adverse effects of steroids are time and dose dependent. Adjuvant therapies are therefore used to provide a steroid-sparing effect. Conventional adjuvants include various immunosuppressive adjuvants such as Azathioprine, Mycophenolate, Methotrexate, Cyclophosphamide, Cyclosporine; and anti-inflammatory agents like gold, Dapsone and many others. Unfortunately these medications are often associated with significant toxicities. Though the majority of the patients will ultimately respond to these therapies, a few patients develop recalcitrant disease.[1]

Over the years, advances have been made to expand therapeutic armamentarium for pemphigus. Emerging therapies include i.v. immunoglobulin, plasmapheresis, immunoadsorption, extracorporeal photochemotherapy, rituximab, TNF-antagonist and other experimental therapies such as Desmoglein-3 peptides.[3]

Laser at low power has been used very effectively in the treatment of oral lichen planus, leukoplakia, aphthous ulcers and even oral manifestations of HIV.[4–8] So many different lasers, including surgical lasers such as argon, Nd: YAG, diodes and CO_2_, seem to have a stimulative/ regulative effect on tissue that encompasses pain relief and wound healing.[9] It has been suggested that use of CO_2_, which has high coefficient of absorption in water, is very suitable for soft-tissue applications. Furthermore, at low power it supplies direct biostimulative light energy to body’s cells, leading to increased ATP production and increased cellular metabolism. This is clinically important in wound healing.[10] The effect of laser light is usually localized at the treatment site however there can be more generalized systemic effects.[9]

The outcome in our cases suggests that CO_2_ laser may be an effective treatment option for recalcitrant pemphigus vulgaris. In our experience, laser irradiation provides pain relief and improved wound healing, and lesions usually do not recur. However, further studies with randomized controlled trials are required to establish the efficacy of laser in the management of oral pemphigus vulgaris patients.
